# Increased human defensine levels hint at an inflammatory etiology of bisphosphonate-associated osteonecrosis of the jaw: An immunohistological study

**DOI:** 10.1186/1479-5876-9-135

**Published:** 2011-08-15

**Authors:** Philipp Stockmann, Falk Wehrhan, Stephan Schwarz-Furlan, Florian Stelzle, Susanne Trabert, Friedrich W Neukam, Emeka Nkenke

**Affiliations:** 1 Department of Oral and Maxillofacial Surgery, University of Erlangen-Nuremberg, Erlangen, Germany; 2 Department of Pathology, University of Erlangen-Nuremberg, Erlangen, Germany

**Keywords:** antimicrobial peptide, bisphosphonate-associated osteonecrosis, osteoradionecrosis, human beta defensins, innate immunity

## Abstract

**Background:**

Human β-defensins (hBD) are antimicrobial peptides that are an integral part of bone innate immunity. Recently, it could be shown that expression of hBD-1, -2 and -3 were upregulated in cases of osteomyelitis of the jaws. In order to gain insight into the possible impairment of hBD metabolism in bisphosphonate-associated osteonecrosis of the jaws (BONJ), the present exploratory study was designed so as to determine the qualitative and quantitative expression of afore mentioned hBDs in BONJ and infected osteoradionecrosis (ORN), both of which represent inflammatory bone diseases.

**Methods:**

Bone samples were collected from patients with BONJ (n = 20) and ORN (n = 20). Non-infected healthy bone samples (n = 20) were included as controls. Immunohistological staining in an autostainer was carried out by the (Strept-ABC)-method against hBD-1,-2,-3. Specific positive vs. negative cell reaction of osteocytes (labeling index) near the border of bony resection was determined and counted for quantitative analysis. Number of vital osteocytes vs. empty osteocytes lacunae was compared between groups.

**Results:**

hBD-1,-2 and -3 could be detected in BONJ as well as ORN and healthy bone samples. Immunoreactivity against hBD-2 and -3 was significantly higher in BONJ than in ORN and healthy jaw bone samples. Number of empty osteocyte lacunae was significantly higher in ORN compared with BONJ (*P *= 0.001).

**Conclusion:**

Under the condition of BONJ an increased expression of hBD-1,-2,-3 is detectable, similarly to the recently described upregulation of defensins in chronically infected jaw bones. It remains still unclear how these findings may relate to the pathoetiology of these diseases and whether this is contributing to the development of BONJ and ORN or simply an after effect of the disease.

## Background

Bisphosphonates are an important component of treatment in metastatic bone disease and the management of osteoporosis. An increasing number of reports have associated the use of bisphosphonates with the occurrence of osteonecrosis of the jaw.

The clinical symptoms of bisphosphonate-associated osteonecrosis of the jaw (BONJ) are rather similar to the lesions seen in patients with infected osteoradionecrosis (ORN) [1]. The lesions are surrounded by inflammatory soft tissue reactions and show symptoms and radiological signs of bone sequestration and/or osteomyelitis [2]. Microorganisms like Actinomyces spp. seem to play an etiological role in the development of both ORN and BONJ [3-5].

Defensins are antimicrobial peptides that are an integral part of innate and antigen-specific acquired immunity [6,7]. These small cationic and cysteine-rich peptides (3.5 to 6.5 kDA) have the potency to disrupt membranes and interfere with intracellular functions of various gram-positive and gram-negative bacteria as well as fungal and encapsulated viral pathogens [8]. Even the potential role of defensins in pathogenesis of oral cancer is under discussion [9,10]. To date, various numbers of defensins subdivided into α- and β- and θ-defensins have been discovered in humans [11,12]. They are characterized and distinguished owing to their sequence homology and disulfide pairing. Human β-defensins (hBD) -1, -2 and -3 have a broad spectrum antimicrobial activity and structural similarities [13]. They are mainly produced by epithelial cells, but quite recently Warnke and co-workers adduced evidence that they are expressed by osteocytes in jaw bone as well. These findings might explain the relatively rare occurrence of osteomyelitis after exposure of jaw bone e.g. after surgical dentoalveolar procedures with exposure of jaw bone to the oral cavity and hint to the important role of these peptides in the pathophysiological mechanism of inflammatory jaw bone diseases [14].

As elevated human hBD -1, -2, -3 levels have been detected in osteomyelitis of the jaw, this leads us to the hypothesis that an impaired hBD expression in the bone might contribute to the development of BONJ or other inflammatory jaw bone diseases like the infected osteoradionecrosis (ORN). Therefore, it was the aim of the present exploratory study to prove and quantify β-defensin expression in BONJ and to compare it with ORN and healthy jaw bone as a control.

## Methods

After approval by the ethical committee of the University of Erlangen-Nuremberg bone biopsies of patients suffering from BONJ (n = 20) and ORN (n = 20) as well as control samples (n = 20) of healthy jaw bone were used for evaluation. All bone samples were harvested in the molar region of the mandible. The samples of BONJ and ORN comprised non-necrotic bone adjacent to necrotic zones. Controls were surplus of resected uninfected bone during orthognathic surgery. All patients had been informed about this study and gave their informed consent for participation.

The average age at surgery was 70 ± 11 years in the BONJ group, 59 ± 8 years in the ORN group and 46 ± 13 years in the control group.

BONJ was defined as an area of exposed bone in the maxillofacial region that did not heal within eight weeks of identification by a health care provider, in a patient who was receiving or had been exposed to a bisphosphonate and had not had radiation therapy to the craniofacial region [15,16]. 6 patients in the BONJ group suffered from metastatic prostate cancer, 8 patients had breast cancer and 6 patients had plasmocytoma. 13 patients received IV zoledronate and 6 patients pamidronate and 1 patient received ibandronate after zoledronic acid on a monthly basis. The mean duration of bisphosphonate therapy was 34.3 ± 23.5 months before surgery was carried out. All patients in this group underwent osteotomy of the necrotic bone followed by primary wound closure [17].

According to Marx, ORN is defined as exposed irradiated bone tissue that fails to heal over a period of three months without a residual or recurrent tumor [18]. Clinical signs of inflammation and bacterial superinfection lead to a diagnosis of infected ORN.

**Figure 1:**
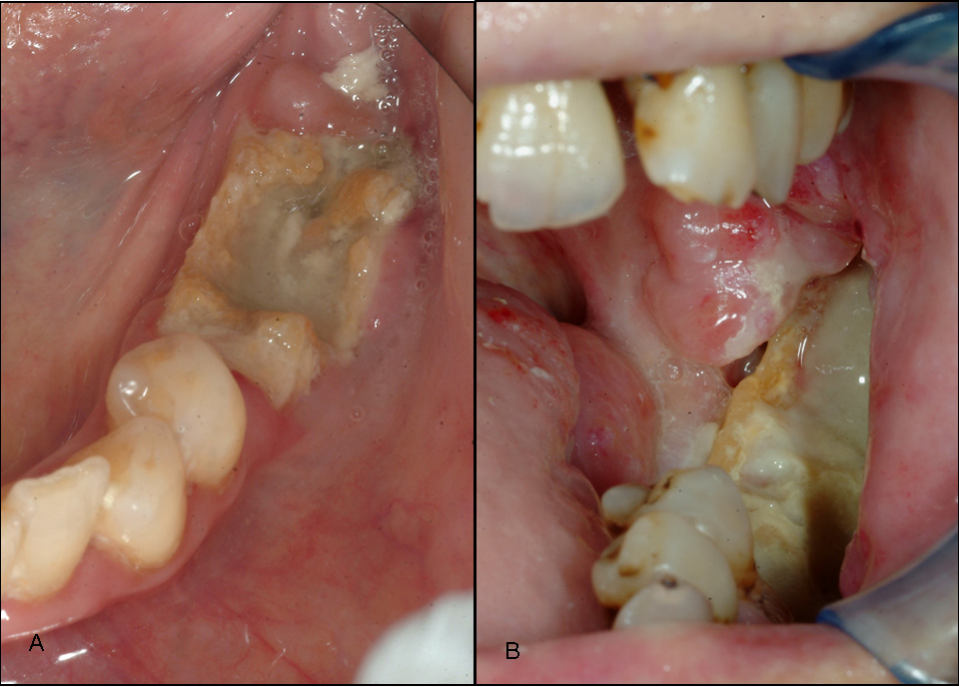
**The clinical picture of bisphosphonate-associated osteonecrosis of the jaw is rather similar to the lesions seen in patients with infected osteoradionecrosis**. Clinical pictures of a stage 2 bisphosphonate-associated osteonecrosis of the jaw (A) and infected osteoradionecrosis (B). Bone samples of these clinical manifestations were included in the study.

Bone samples of BONJ and ORN were only included in the study when the region of exposed bone showed signs of infection as evidenced by pain and erythema with or without purulent drainage (stage 2 of BONJ [15]) and the patients were not under permanent medication with steroids (Figure 1).

### Immunohistochemistry

Bone biopsies were fixed in neutral 4% Formalin solution. Afterwards, the samples were decalcified in a 25% ethylenediaminetetraacetic acid (EDTA) solution (pH 7.4). The decalcification lasted 10 days and the EDTA solution was changed several times during the process. The dehydration procedure was performed in an ascending alcohol sequence at room temperature in a dehydration unit (Shandon Citadel 1000, Shandon GmbH, Germany). Paraffin-embedded bone samples were sectioned in cuts of 4 μm thickness with a standard microtome (Leica RM 2165^®^, Leica Microsystems, Nussloch GmbH, Germany). Subsequently, the surface of the samples was blocked to prevent unspecific staining using a serum-free protein block (DAKO Diagnostics GmbH, Germany).

Immunohistological staining was obtained for detection of expression of hBD-1, -2, and -3 with the Strepavidin-Biotin-Peroxidase-complex (Strept-ABC)-method performed for all bone samples with an autostainer (Autostainer plus^®^, DakoCytomation, Dako Deutschland GmBH, Germany). To deparaffinize the slices we washed them in Xylol and then cooked them for 15 min in EDTA-buffer (Dako Retrieval Puffer, pH 9,0) to uncover the relevant antigens. We applied 3% H_2_O_2 _to block endogeneous peroxidase. The slides were washed in Tris-Buffered Saline (TBS) and incubated with rabbit antisera to hBD-1 (Biologo, DEF01-A, Kiel, Germany, dilution 1:500), hBD-2 (DEF02, dilution 1:250) and hBD-3 (DEF03-S, dilution 1:500) as well as pre-immune serum as negative control. Further processing of the Strept-ABC method was carried out according to the manufacturer's manual (Dako, Hamburg, Germany). Finally, the samples were stained with Hematoxylin-Eosin (Dako S 3301) for light microscopic evaluation. Negative controls without primary antibody were passed by in each cycle to verify antibody specificity.

### Qualitative and quantitative analysis

Qualitative and quantitative analyses were performed for the absence of osteocytes in the osteocyte lacunae next to each Kwire track as a measure of bone necrosis. For quantification of hBD-1 through three expressions the immunostained slices were analyzed and digitized with a light microscope (Axioscope^® ^Zeiss, Jena, Germany). Regions of interest (ROI) were bone areas in spongy bone which showed equal bone trabecular and bone marrow cells. Three visual fields per section for each sample were digitized with a CCD camera. In a 400-fold magnification the analyzing software enabled cells inside an ROI to be digitally marked, and measurement parameters were determined by means of Bioquant Osteo^® ^software V7.10.10 (Nashville, USA). As a sign of bone necrosis the numbers of empty osteocytes lacunae were related to the number of total count of osteocytes inside the ROI. The labeling index was defined as the ratio of stained osteocytes vs. total number of osteocytes/osteocytes lacunae inside the ROI. The intensity of immunostaining was not considered for the labeling index.

### Statistics

For statistical analysis, group means and standard deviations were calculated for each parameter with SPSS software (version 16; SPSS Inc., Chicago, USA). Data were compared with the Mann-Whitney-U-Test. A *P*-value < 0.05 was considered statistically significant.

## Results

In ORN it was evident that there were 75.0% empty osteocyte lacunae whereas BONJ had only 24.8% empty lacunae inside the ROI. The healthy bone samples showed 2.4% empty osteocyte lacunae. The number of vital osteocytes was significantly higher in BONJ than ORN (*P *= 0.001).

Specific immunoreactivity was able to identify the presence of hBD-1, -2 and -3 within jaw bone biopsies in all tested groups. Expression is especially prominent in osteoblasts and the osteocytes included in woven bone while the resting osteocytes in lamellar bone are negative.

### Human β-defensin-1 (Figure 2)

**Figure 2:**
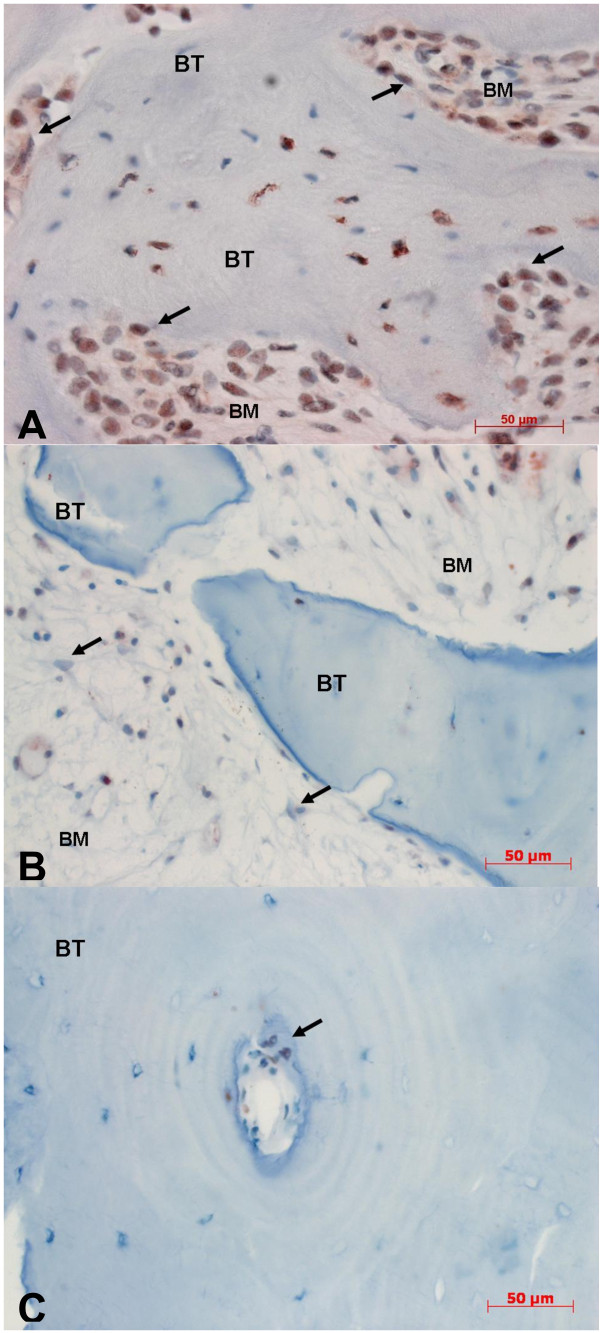
**Immunohistochemical staining against hBD-1 (400-fold magnification)**. **A: **BONJ. Increased immunoreactivity can be detected within bone marrow area (BM), mostly in bands along the endosteal cell lines (arrows). Inside mineralized bone trabecula (BT) immunostaining demonstrates nuclear expression of hBD-1. **B: **ORN. Immunoreactivity is barely visible in cytoplasm of stromal cells. Specific cells for diagnosis of ORN in terms of radicytes (arrows) were traceable. **C: **Healthy bone samples. Detailed enlargement of mineralized area (BT) of controls shows a Haversian channel (arrow) in which positive nuclear staining is seen only along the endosteal cell line. Immunoreactivity of osteocytes is negative in this sample.

BONJ samples showed high immunoreactivity in stromal cells including rims of osteoblasts along the endosteal cell lines. Numerous osteocytes in the mineralized bone trabeculae showed specific positive cell reactions.

Besides typical cell changes (radiocytes) in ORN the immunoreactivity of osteocytes against hBD-1 was barely visible and was detectable mostly in stromal cells. Non-viable bone was demonstrated by lack of nucleoli in bone lacunae. Large parts of the bone marrow showed fibronecrotic lesions and fibrosis without any immunoreactivity against hBD-1.

**Figure 3:**
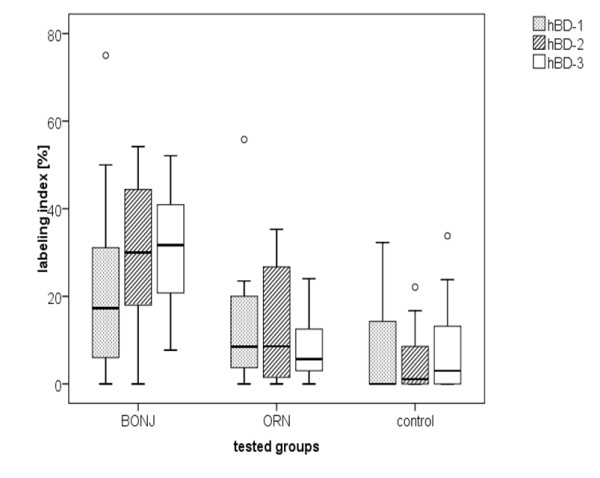
**Immunoreactivity against hBD-2 and -3 was significantly higher in bisphosphonate-associated osteonecrosis of the jaw than in infected osteoradionecrosis and healthy jaw bone samples**. Value distribution of hBD-1 trough 3 expression in bone shown as boxplots divided into groups. The labeling index was defined as the ratio of stained osteocytes vs. total number of osteocytes and osteocyte lacunae inside the ROI. Outliers are marked as circles.

Healthy bone showed only smooth positive cell reaction mostly in the vicinity of blood vessels and bone marrow. The average value of the labeling index revealed that positive cell reaction to hBD-1 was slightly higher in BONJ (22.3 ± 20.3%) than in ORN (7.2 ± 10.4%) and healthy jaw bone samples (12.8 ± 14.8%). These differences were not statistically significant (Figure 3).

### Human β-defensin-2 (Figure 4)

**Figure 4:**
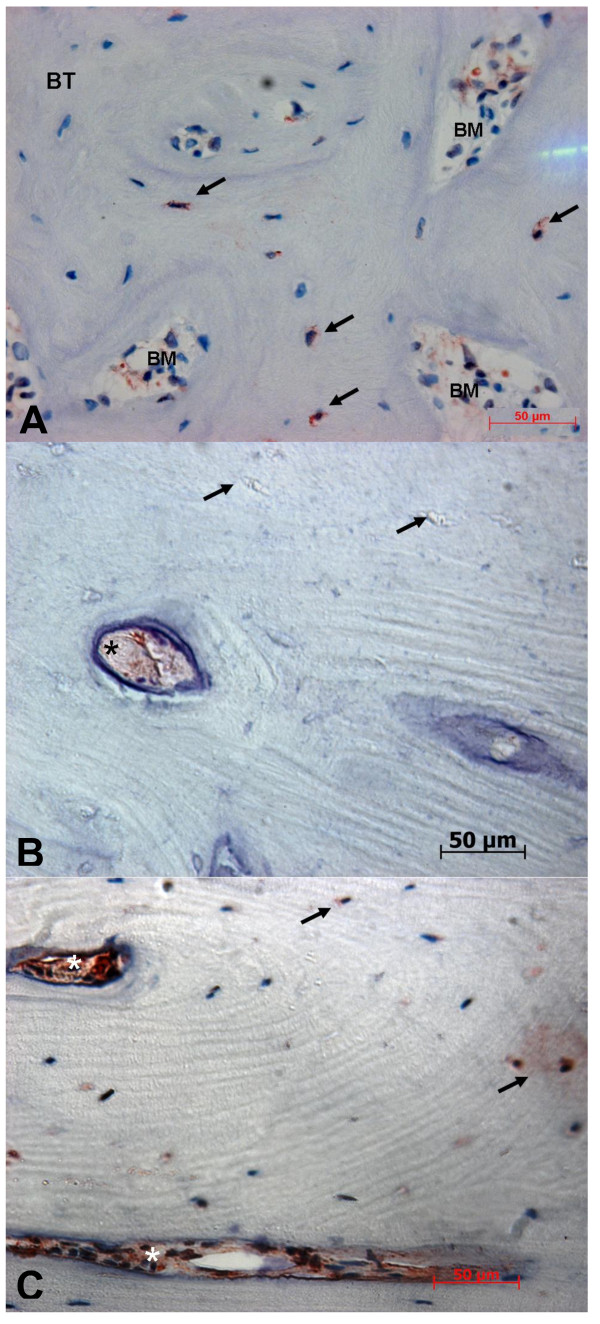
**Immunohistochemical staining against hBD-2 (400-fold magnification)**. **A: **BONJ. Smooth positive staining could be detected along the endosteal cell lines. Inside bone trabecula (BT) scattered positive cell reactions of osteocytes are visible, which indicate expression of hBD-2. **B: **ORN. Negative empty osteocytes lacunae (arrow) and enhanced positivity in the endosteal cell line (star) are evident. **C**: Healthy bone samples. Partial positivity of osteocytes (arrow) and weak cytoplasmic positivity of stromal cells (stars) within the bone marrow area (BM) are visible.

Intensity of immunostaining in hBD-2 was weaker compared with hBD-1 in all groups.

Inside bone trabecula of BONJ samples sufficient immunoreactivity could be often found in the cytoplasm of osteocytes and stromal cells, which indicates expression of hBD-2 in these cells. Smooth positive staining could be detected along osteoblasts lined up at the endosteal cell lines. Moreover, the highest extensive immunoreactivity was visible in areas of bone marrow, which showed infiltration with polymorphonuclear leukocytes, indicating a reaction to acute inflammation.

In ORN samples osteocytes had mainly negative cell reactions to hBD-2, and enhanced positivity was observed only in osteoblasts near the endosteal cell line. Similarly, healthy bone showed weak cytoplasmic positivity of osteocytes and stromal cells.

Quantitative analysis revealed an average labeling index of 29.2 ± 16.4% in BONJ samples. Significantly lower values for the labeling index could be observed in healthy jaw bone (12.3 ± 12.5%, *P *= 0.017) and smallest values in ORN (5.0 ± 7.0, *P *= 0.002) (Figure 3).

### Human β-defensin-3 (Figure 5)

**Figure 5:**
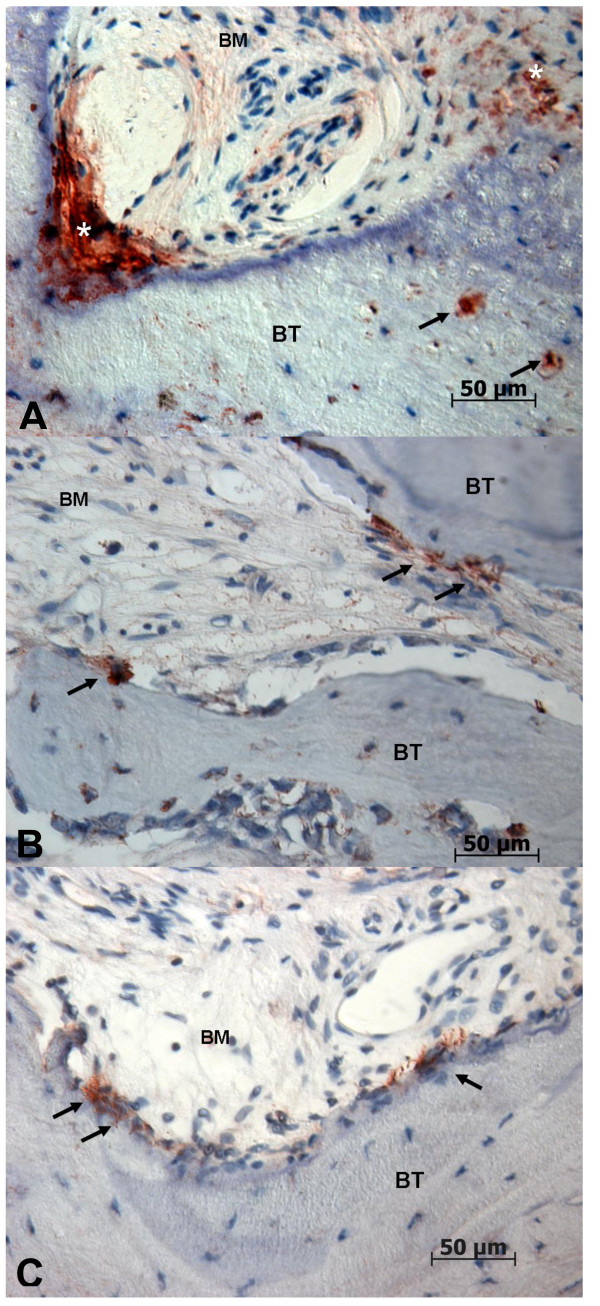
**Immunohistochemical staining against hBD-3 (400-fold magnification)**. **A:**. BONJ. Immunoreactivity could be detected along the endosteal cell lines (stars). Inside bone trabecula (BT) numerous positive cell reactions of osteocytes are visible (arrows), which indicate expression of hBD-3. BM, bone marrow area. **B: **ORN. Strong positivity in osteoblasts could be detected along the endosteal cell line (arrows). Inside mineralized bone trabecula (BT) only weak positivity in osteocytes is evident. **C: **Healthy bone controls. Positive staining could be detected along the endosteal cell lines (arrows) with moderate positivity of osteoblasts, which indicates the presence of hBD-3. Inside bone trabecula (BT) a no positive osteocytes are visible.

There was distinctive immunoreactivity against hBD-3 in all groups compared with hBD-1 and -2. The highest intensity of immunostaining was detectable beside the border of bone marrow and mineralized bone. Bands of osteoblasts at the endosteal cell line showed intensive dyeing.

As regards the labeling index, the average value for ORN (8.1 ± 10.3%) and healthy bone biopsies (8.1 ± 7.4%) were approximately equal compared to the significantly higher measured values for BONJ (30.7 ± 16.4, p < 0.05) (Figure 3).

## Discussion

To date the etiopathogenesis of BONJ is not sufficiently clarified. Different hypotheses concerning the pathophysiology of BONJ are to be found in the literature: The inhibition of osteoclast and osteoblast activity followed by an impaired bone turnover with compromised bone healing [19,20], an inhibition of endothelial cells with impaired intraosseous angiogenesis, mucosal damage secondary to toxic exposure of the bone [21-23] and the infectious-immune hypothesis with impaired immune defense at the mucosal barrier [12,21,22]. All hypotheses could not yet explain the rare occurrence of BONJ and its restriction mainly to the jaws.

Based on the infectious-immune hypothesis and on the knowledge that hBDs are expressed in osteogenetic cell lineages, the main focus of this exploratory study was to determine the expression level of antimicrobial peptides in BONJ, so as to proof the hypothesis, that there is a possible impairment, which could affect susceptibility to BONJ.

As part of innate immunity, antimicrobial peptides like defensins seem to play an important role in protection of oral cavity integrity against invasion by microbes [24]. β-defensin exhibits a bactericidal effect on pathogens that is based on an inhibition of cell proliferation [25] and extracellular matrix production [8] and the modulation of cellular immune responses [26]. The localization of hBD-1-3 in oral mucosa has been confirmed at protein and mRNA levels [24].

Recently it was shown that hBD-1, -2 and -3 are expressed in chronically infected as well as healthy jaw bone [14]. Subsequently, Kraus and coworkers could demonstrate that hBD-1, -2, -3 were expressed in osteoblast-like MG63 cells *in vitro*. Moreover, they could provide evidence that hBD-2 stimulates their proliferation and hBD-2 and -3 positively affected their differentiation processes [27].

To date, the detailed pathways regulating the expression of human β-defensins are not completely understood. It seems that hBD-1 may be modulated by inflammation, while hBD-2 and hBD-3 are expressed by cells upon stimulation with proinflammatory cytokines and by microorganisms [24]. hBD-1 can be induced and upregulated by lipopolysaccharides (LPSs), heat-inactivated Pseudomonas eruginosa and interferon gamma (IFN-γ). hBD-2 expression is induced in response to gram- and gram+ bacteria as well as Candida albicans [28]. In contrast with hBD-2, upregulation of hBD-3 expression in keratinocytes was observed in the presence of inflammatory proteins like transforming growth factor alpha (TGF-α) and insulin-like growth factor 1 (IGF-1) [29].

There are data to indicate that nitrogen-containing bisphosphonates affect the function of cells of both innate and acquired immunity. In particular, these agents have a profound effect on differentiation and maturation of human myeloid dendritic cells (DC) [30]. Interestingly, both hBD-1 and -2 seem to possess immunoregulatory activity as well, by chemoattraction of immature dendritic cells and memory T cells through interaction with beta chemokine receptor [31].

In addition nitrogen-containing bisphosphonates have been shown to augment the allostimulatory activity of DC on naive CD4^++ ^and CD45^+ ^T cells in terms of their proliferation and interferon-γ production [32]. There is evidence that hBD-3 expression is inducible by interferon-γ [33], which might be the reason why hBD-3 showed the highest immunoreactive values in BONJ samples in our study.

Also, it has been shown that the activation of Vγ9Vδ2 T cells by aminobisphosphonate drugs results in a massive release of cytokines and chemokines that may induce expression of defensines. Moreover, that soluble factors released by aminobisphosphonate -stimulated Vγ9Vδ2 T cells activate granulocytes by inducing their chemotaxis, phagocytosis, and alpha-defensins release [34].

A lack of induction of osteoblast-derived hBD-2 in the presence of immunosuppressive drugs, which are frequently used in chronic inflammatory joint diseases, is assumed to be responsible for the increased susceptibility of these patients to bone and joint infection [35].

So far, no studies are available that have determined the expression of defensins in BONJ. Therefore, the present study was conducted to determining the expression of human β- defensins in BONJ quantitatively. Because of a number of similar clinical and pathological features, samples of infected osteoradionecrosis were also examined in the present study. Although both conditions are related to bacterial infection (e.g. Actinomyces) and they share similar clinical symptoms, there are differences in their histological appearance. BONJ shows elements of osteomyelitis and it is not directly comparable to osteoradionecrosis of the mandible [12]. In particular, areas of active acute inflammation with the presence of inflammatory cells were seen in peripheral areas, where organized bacterial biofilms were present [36,37].

To the best of our knowledge this is the first report on the expression analysis of hBD in BONJ bone samples. Our hypothesis that hBD expression is hindered in BONJ bone samples could not be confirmed in the present study. However, the results reveal that immunoreactivity for antimicrobial peptides hBD-1, hBD-2 and hBD-3 in jaw bone biopsies of BONJ can be found on a regular basis. The results indicate that jaw bone samples harvested from BONJ are still able to express defensins on a higher level than healthy uninfected jaw bone. This result points out, that there is still an unimpaired metabolic reaction in BONJ bone samples due to an infection stimulus. In contrast, the expression of human β-defensins in ORN was significantly reduced. Therefore, it seems that bone affected by BONJ does not exclusively show characteristics of necrotic bone like ORN samples, but behaves in a similar fashion to that described previously for bone suffering from bacterial infection [14]. Some authors have already pointed to the role of infection in BONJ. Hansen and colleagues showed that 93.5% of patients suffering from BONJ also had a superinfection of Actinomyces israelii [1,4]. Sedghizadeh and colleagues examined bony sequesters of BONJ by electron microscopy and identified various species of the genus Fusobacterium, bacillus, actinomyces, staphylococcus, streptococcus, Selenomonas, and three different morphotypes of treponemes or spirochetes, which were organized in complex biofims [36]. Staining of hBD-3 seemed to be distinctly more intense in all samples compared to hBD-1 and -2. This may indicate that there is an intrinsic basal level of hBD-3 expression that is independent on exposure to bacterial stimuli. Similar results that were seen in healthy periodontal tissues and tissue samples of healthy bone suggest a potentially important protective role of defensins in the host immune response to infection by oral pathogens [38,39].

At the moment, however, it is not clear if infection is a major etiological factor for BONJ or just a sequela of this disease. It seems that BONJ is a multifactorial process resulting from an alteration in bone homeostasis, inhibition of angiogenesis and, in particular, bacterial risk factors [20,21,40].

While these are interesting findings it is not clear how these results may relate to the pathoetiology of BONJ and ORN and whether this is contributing to the development of the diseases or simply an after effect of the disease. Additionally, the methodology of the presented study leads to no conclusion whether the expression of hBD-1 through 3 is associated with the degree of inflammation, the presence or the amount of bacteria or the severity of BONJ and ORN. However, the increased expression of human β-defensins in bone samples of BONJ can be interpreted as a sign of unimpaired metabolic activity and can therefore be seen as a reaction of vital bone to microbial invasion. In this context, the study could demonstrate a significant difference between BONJ and ORN concerning their potency in immunological response. The question that remains still unanswered is whether the defensins retain their full functionality in the bisphosphonate-laden bone. In addition, the present study provides no data regarding the regulation or induction process of hBD in BONJ and ORN.

Future research needs to clarify whether the increased expression of β-defensins in BONJ suggests that bone infection is the crucial point in BONJ while osteonecrosis only accompanies the disease. It has been proposed previously that BONJ should rather be termed bisphosphonate-associated osteomyelitis of the jaws [12]. Hence, further studies should focus on the discovery of the detailed function of the three hBDs in innate and adaptive immune system especially in the jaw bone and the possible impact of bisphosphonates on their immunological pathway. The results of the study, which hint at an inflammatory etiology, can further help to optimise preventive measures and existing treatment regimes, e.g. avoidance of extended exposition of bisphsophonate-laden jaw bone to the oral cavity, the importance of supportive application of antibiotics and strengthen of the immune system by influencing the local immune defence.

## Conclusions

Under the condition of BONJ an increased expression of hBD-1,-2,-3 are detectable, similarly to the recently described upregulation of defensins in chronically infected jaw bones. It remains still unclear how these findings may relate to the pathoetiology of BONJ and whether this is contributing to the development of BONJ or simply an after effect of the disease.

Future research should focus on evolving the specific role of hBDs in the innate and adaptive immune system of the bone and whether there is a possible impairment of their antimicrobial activity under the influence of bisphosphonates. Thereby, knowledge could be derived regarding the understanding of the etiopathogenesis and subsequently the prevention and treatment of BONJ.

## Competing interests

The authors declare that they have no competing interests.

## Authors' contributions

PS was responsible for the conduction of study, built the hypothesis, established and conducted the methods and analytic procedures and wrote the manuscript. SS and FW interpreted the histopathological samples and performed the immunohistochemistry analysis. ST participated in the design of the study and performed immunohistochemistry. FS worked on the statistical analysis. FWN have given final approval of the version to be published. EN interpreted the data and revised the manuscript. All authors read and approved the final manuscript.
